# Thickness Mapping and Layer Number Identification of Exfoliated van der Waals Materials by Fourier Imaging Micro-Ellipsometry

**DOI:** 10.1021/acsnano.2c12773

**Published:** 2023-05-08

**Authors:** Ralfy Kenaz, Saptarshi Ghosh, Pradheesh Ramachandran, Kenji Watanabe, Takashi Taniguchi, Hadar Steinberg, Ronen Rapaport

**Affiliations:** † Racah Institute of Physics, The Hebrew University of Jerusalem, Jerusalem 9190401, Israel; ‡ Research Center for Functional Materials, National Institute for Materials Science, 1-1 Namiki, Tsukuba 305-0044, Japan; § International Center for Materials Nanoarchitectonics, National Institute for Materials Science, 1-1 Namiki, Tsukuba 305-0044, Japan

**Keywords:** spectroscopic ellipsometry, van der Waals materials, mechanical exfoliation, hexagonal boron nitride, transition metal dichalcogenides, thickness mapping, modeling

## Abstract

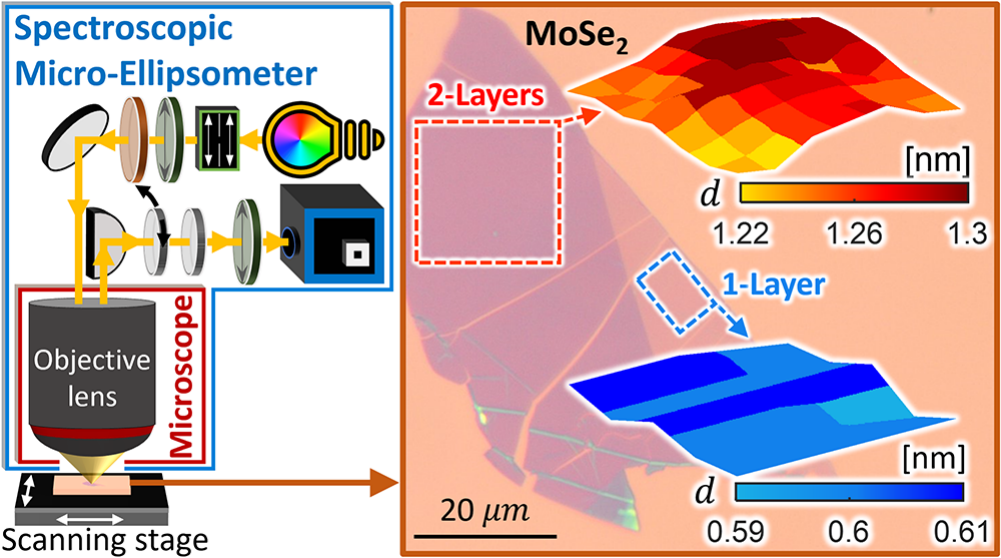

As performance of van der Waals heterostructure devices is governed by the nanoscale thicknesses and homogeneity of their constituent mono- to few-layer flakes, accurate mapping of these properties with high lateral resolution becomes imperative. Spectroscopic ellipsometry is a promising optical technique for such atomically thin-film characterization due to its simplicity, noninvasive nature and high accuracy. However, the effective use of standard ellipsometry methods on exfoliated micron-scale flakes is inhibited by their tens-of-microns lateral resolution or slow data acquisition. In this work, we demonstrate a Fourier imaging spectroscopic micro-ellipsometry method with sub-5 μm lateral resolution and three orders-of-magnitude faster data acquisition than similar-resolution ellipsometers. Simultaneous recording of spectroscopic ellipsometry information at multiple angles results in a highly sensitive system, which is used for performing angstrom-level accurate and consistent thickness mapping on exfoliated mono-, bi- and trilayers of graphene, hexagonal boron nitride (hBN) and transition metal dichalcogenide (MoS_2_, WS_2_, MoSe_2_, WSe_2_) flakes. The system can successfully identify highly transparent monolayer hBN, a challenging proposition for other characterization tools. The optical microscope integrated ellipsometer can also map minute thickness variations over a micron-scale flake, revealing its lateral inhomogeneity. The prospect of adding standard optical elements to augment generic optical imaging and spectroscopy setups with accurate *in situ* ellipsometric mapping capability presents potential opportunities for investigation of exfoliated 2D materials.

## Introduction

Ever since mechanical exfoliation of single-layer graphene from graphite,^1^ the two-dimensional (2D) inventory has expanded considerably to include hexagonal boron nitride (hBN) and transition metal dichalcogenides (TMDs) with strong in-plane and weak out-of-plane van der Waals (vdW) molecular bonds. In such vdW layered structures, thickness (and thus layer number) is a crucial parameter for realizing many exotic effects, such as superconductivity,^2^ exciton and exciton-polariton Bose–Einstein condensation,^3−5^ generalized Wigner crystals,^6^ tunnel barriers^7,8^ and exotic correlated states.^9−20^ Yet, an accurate, fast and noninvasive method capable of performing *in situ* lateral mapping of angstrom-level thicknesses is still missing. To date, such layer numbers and their sub-nanoscale thicknesses are primarily estimated by optical microscopy, and subsequently evaluated by Raman spectroscopy or atomic force microscopy (AFM).^21,22^

The effective thickness of a flake depends on multiple factors such as presence of physisorbed organic molecules and other adsorbents,^23,24^ air gaps at substrate–flake interfaces,^25^ as well as exerted pressure that can alter bond length and consequently the thickness.^26^ These along with differences in gradients of the attractive and lateral forces on the material and the substrate,^27^ and anomalies due to tip–sample interactions^28,29^ are common causes for misinterpreting the thickness values using an AFM instrument, which might also be invasive especially in the more-accurate contact mode.^30^ Optical microscopy provides a simpler way for estimating the exfoliated flake’s layer number, and not the actual thickness, based on the color contrast.^21,31^ However, it involves uncertainties at times due to dependence on human cognition. Another challenge faced by optical microscopy is the high transparency of mono- and few-layer hBN which makes their identification a challenging proposition (a maximum of 2.5% white-light contrast is achievable for monolayer hBN).^32,33^ As such, the layer number of an hBN flake is typically characterized by Raman spectroscopy, as its thickness alters the width and position of phonon modes in the Raman spectra.^32,33^ However, the signal and its dependence on thickness is extremely weak, thus requiring long integration times while also varying considerably among samples.^32,33^ As for graphene and TMDs, Raman spectroscopy is widely accepted as the ideal method for distinguishing among layer numbers,^22,34,35^ although exposing the material to varying laser powers might result in increase in the lattice temperature by strong optical absorption, which corresponds to shifts in the Raman frequencies.^36,37^

Spectroscopic ellipsometry offers a noninvasive yet highly sensitive optical technique for accurate thickness measurements, relying on the incident light to penetrate through the partially absorbing thin-film.^38^ The technique can also extract the optical properties from 2D vdW materials.^39,40^ Spectroscopic ellipsometry was successfully used in measuring various thicknesses of highly transparent hBN with angstrom-level precision, from monolayers^33^ to much thicker flakes (>100 nm).^41^ However, current spectroscopic ellipsometers are limited to off-axis illumination for oblique angles of incidence, and even with integrated focusing optics, they cannot achieve a spot size smaller than ∼50 μm,^42^ thus rendering them unsuitable for mapping smaller lateral dimensions which are typical of exfoliated vdW flakes.

To counter this issue, imaging ellipsometers integrate optical microscopy by addition of an objective lens and a 2D detector array to their hardware, increasing the lateral resolution to a few microns.^41,43−48^ However, this method is constrained to a single wavelength at a single incidence angle at-a-time measurements, resulting in very long data acquisition times for spectrally and angularly resolved information,^49^ thus also requiring a very stable sample. Acquisition at multiple angles of incidence is especially important for multilayered structures since each angle traverses a different optical path, providing unique ellipsometric information that is crucial for optimizing sensitivity to the unknown parameters.^38,50^

In this paper, we perform spectroscopic ellipsometry measurements on exfoliated micron-scale vdW flakes with our recently developed Fourier imaging spectroscopic micro-ellipsometry method.^51^ Our spectroscopic micro-ellipsometer (SME) integrates spectroscopic ellipsometry into generic optical microscopy configuration, working in an on-axis configuration with a sub-5 μm lateral resolution (one order-of-magnitude higher compared to focused-beam spectroscopic ellipsometers).^42,52^ Its high data acquisition rate allows recording broadband ellipsometric data at multiple angles of incidence simultaneously at a given lateral position within a few seconds, making it at least three orders-of-magnitude faster compared to imaging ellipsometers, improving the practically achievable level of sensitivity and accuracy in process.

Utilizing these advantages of the SME, we demonstrate highly accurate, angstrom-level precise thickness mapping and consequent layer number identification of exfoliated vdW flakes from different genres, including conductive graphene, various semiconductor TMDs and wide band gap dielectric hBN. Successful ellipsometric identification of a highly transparent monolayer hBN flake (0.32 nm thick) showcases the high sensitivity of the SME. In particular, we investigate the homogeneity of exfoliated MoSe_2_ mono- and bilayers by accurate mapping of the local thickness variations across their micron-scale surface areas with high lateral resolution. Such mapping of in-plane homogeneity is crucial for estimating thickness variations and deviation in optical properties arising from localized strain, which can be identified by spectroscopic ellipsometry exclusively.

## Results and Discussion

**Figure 1:**
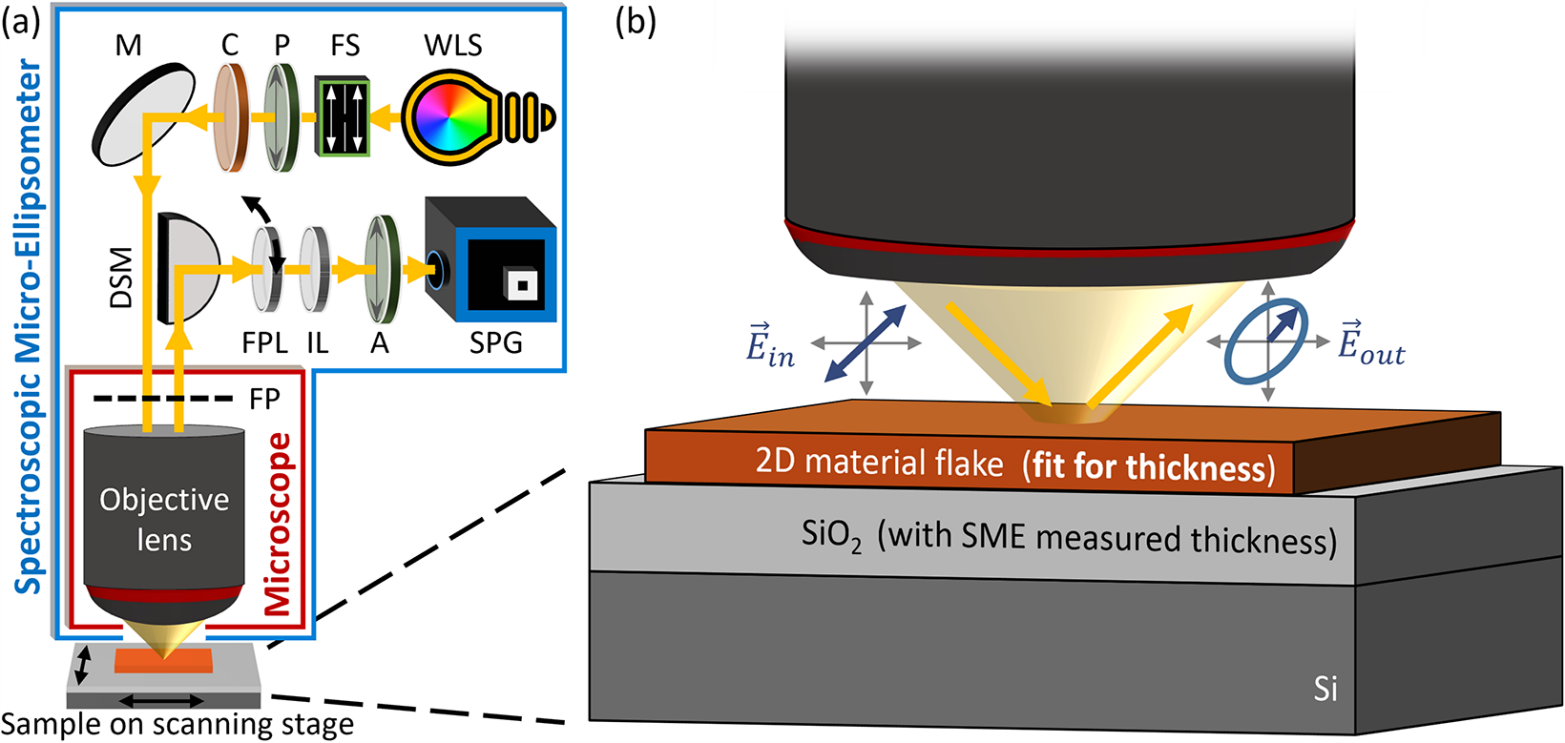
(a) Schematic of the SME. WLS: White light source, FS: field stop, P: polarizer, C: compensator (quarter-wave retarder), M: mirror, DSM: D-shaped mirror, FP: Fourier plane, FPL: Fourier plane lens (on a flip mount - for interchanging between sample-imaging microscopy mode and the Fourier plane imaging ellipsometry mode), IL: imaging lens, A: analyzer (polarizer), SPG: spectrograph with a 2D detector array. (b) Multiple-angle reflection of polarized white light from the sample results in sample-dependent variations on the polarization state per incident angle, namely, transformation of linear polarization  to elliptical polarization . Previously measured nearby SiO_2_ thickness and the optical constants from the literature for the measured 2D material are used in the model for extraction of the flake thickness.

The schematic of the spectroscopic micro-ellipsometer (SME)^51^ and the illustration for the flake measurement are shown in Figure 1. High numerical aperture (NA) objective lenses provide acquisition of more angular data up to higher angles of incidence, together with a better lateral resolution (smaller spot size), thus are preferred for the SME. After locating the flake of interest under the objective lens (NA = 0.9) of the SME in microscopy mode (see Figure 1a), the SME is switched to ellipsometry measurement mode. Measurements are performed first on the substrate just outside the periphery of the flake and subsequently on the flake, obtaining the spectrally and angularly resolved spectroscopic ellipsometry data of both points (or areas in case of mapping experiments). The thickness of the oxide layer in the vicinity of the flake is determined by the SME and is used in the model when fitting for the thickness of the residing flake, as seen in Figure 1b, and is assumed to not fluctuate considerably under the flake (see Supporting Information (SI) section S3). Next, depending on the flake material, the complex refractive index values obtained from refs (53−55) are used in the model, and the thickness of the flake is fitted for. The obtained thickness value of the flake is used to determine the number of layers. Due to different experimental methods used in the literature to extract the optical constants, they might not exactly coincide with those of the flakes measured by the SME. However, these possible deviations in optical constants do not interfere with the ability of the model to predict the number of layers of the measured flakes.

**Figure 2:**
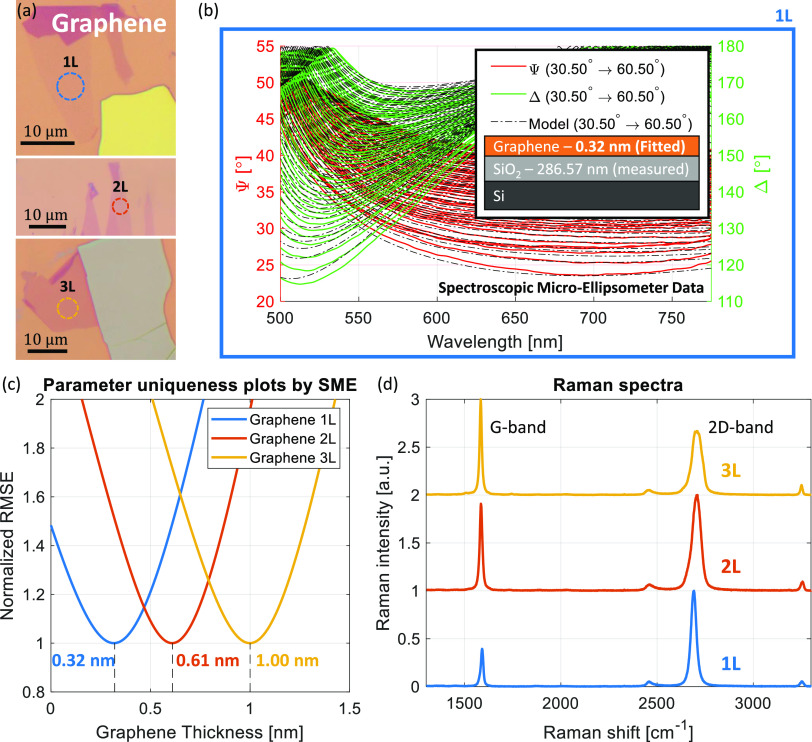
(a) Optical microscope images of monolayer (1L), bilayer (2L) and trilayer (3L) graphene flakes on 285 nm SiO_2_/Si with illustrated 5 μm diameter SME measurement spots. (b) Single-measurement SME data on the monolayer consisting of spectrally and angularly resolved Ψ and Δ values. The model is illustrated in the plot legend, with inputs of SME measured SiO_2_ thickness and graphene optical constants from the literature, resulting in best fit at the graphene thickness value of 0.32 nm. The same procedure is repeated for bilayer and trilayer graphene. (c) The parameter uniqueness plots by the SME for monolayer, bilayer and trilayer measurements pointing to graphene thicknesses of 0.32 nm, 0.61 nm and 1.00 nm, respectively (RMSE: root-mean-square error). (d) The Raman spectra measured on the same flakes confirm their mono-, bi- and trilayer natures measured by the SME.

The optical microscope images of monolayer (1L), bilayer (2L) and trilayer (3L) graphene with illustrated 5 μm diameter SME measurement spots are shown in Figure 2a (these flakes were also used in our previous work^51^). Figure 2b plots the SME data from a single measurement on the monolayer graphene, consisting of ellipsometric parameters Ψ and Δ at 551 wavelength points between 500 and 775 nm, and at 52 different angles of incidence between 30.50° and 60.50°. The change in light polarization reflected from the sample is represented by parameters Ψ and Δ (Ψ is related to the amplitude ratio between the s- and p-components of the polarized light, whereas Δ is the phase difference between them, see ref (51) for more details). Importantly, this whole set of data is acquired with just four exposures (at different measurement polarization settings), in a total measurement time of around 45 s (see Materials and Methods).

The measured oxide thickness by the SME and the complex refractive index of graphene obtained from ref (54) are used in the model to fit for the flake thickness, as shown in Figure 2b for the monolayer graphene. The same procedure is repeated for bilayer and trilayer graphene flakes. Figure 2c shows the parameter uniqueness plots for all three measurements, normalized to their corresponding minimum values. These plots represent the error (in normalized RMSE or root-mean-square error) between the experimental data and the model when the model is scanned for the flake thickness parameter. The global minimum of each curve provides the best fit between the ellipsometric data (i.e., Ψ and Δ) and the model, which for the monolayer occurs at graphene thickness of 0.32 nm. This is in good agreement with the theoretical thickness of 0.34 nm for single-layer graphene.^56^ Similarly, thicknesses of 0.61 and 1.00 nm are obtained for the bilayer and trilayer graphene respectively, again in agreement with the literature.^28^ Each measurement on mono-, bi- and trilayer graphene is repeated 10 times to demonstrate the instrumental accuracy in graphene thickness results. Standard deviations of ∼0.02 nm are obtained for all the three sets of measurements. Finally, the Raman spectra of the same flakes are measured (with laser excitation wavelength of 514.5 nm). Figure 2d plots the normalized and vertically displaced (for better visibility) Raman spectra of the graphene flakes, confirming the findings of the SME. The ∼2.5 peak intensity ratio of the 2D-band to the G-band (*I*_2D_/*I*_G_) and the symmetric 2D-band at ∼ 2690 cm^–1^ with a full width at half-maximum (FWHM) ∼ 33 cm^–1^ provide an exclusive signature for monolayer graphene.^57−59^ Similarly, *I*_2D_/*I*_G_ ∼ 1.1, 0.67 intensity ratios and asymmetric 2D-bands with FWHM ∼ 53, 62 cm^–1^ show the typical features of bilayer and trilayer graphene, respectively.^58−60^

**Figure 3:**
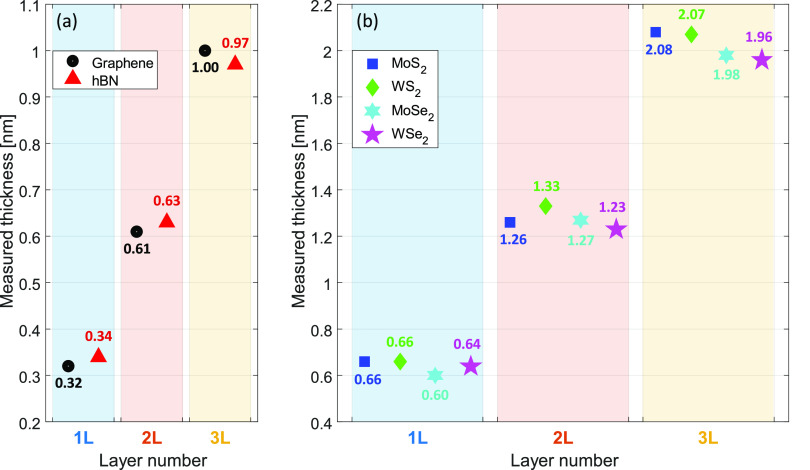
SME derived thicknesses of mono- (1L), bi- (2L) and trilayer (3L) flakes of (a) graphene, hBN, (b) MoS_2_, WS_2_, MoSe_2_ and WSe_2_.

The same procedure performed on graphene is repeated on mono-, bi- and trilayer candidates of hBN, MoS_2_, WS_2_, MoSe_2_ and WSe_2_ (the TMD flakes, measurements and Raman analyses are elaborated in section S1 of the SI). Figure 3 plots a summary of the measured thicknesses for all the exfoliated vdW flakes residing on 285 nm SiO_2_/Si substrates, where the bi- and trilayer flakes are expected to be integer multiples of the monolayer.^61^ As shown, very good agreements with the single-layer thicknesses of 0.32 nm for hBN^62^ and individual values between 0.6 and 0.7 nm for TMDs (MoS_2_ - 0.67 nm,^63,64^ WS_2_ - 0.65 nm,^65^ MoSe_2_ - 0.7 nm^66^ and WSe_2_ - 0.67 nm^63,67^) are found for 1L, 2L and 3L, as in the graphene measurements. All flakes are also analyzed by Raman spectroscopy for their layer numbers, which show good agreement with the SME results (see SI section S1 for TMDs). The thickness errors provided by the fit algorithm for all materials and all number of layers range from ±0.001 nm to ±0.006 nm, two orders-of-magnitude smaller than the final thickness values. As these numbers are too small to be visible on the plot and also to have any effect on the final results, they are considered negligible.

A major aspect in the ellipsometry measurement of a flake’s thickness is understanding the thickness fluctuation of the substrate oxide layer, as the exact oxide thickness value underneath the flake may have an effect on its thickness result through modeling and fitting of the ellipsometric data. This oxide thickness fluctuation was measured in our recent paper^51^ on a same type of silicon wafer used in this work (see Materials and Methods). The maximal fluctuation in the oxide thickness was measured to be ±0.21 nm. The error bars in measured flake thicknesses resulting from this maximum oxide thickness variation are plotted (see Figure S6) and discussed in section S3 of the SI. As seen, they are found to be relatively small and not interfering with the accuracy of layer number identification.

**Figure 4:**
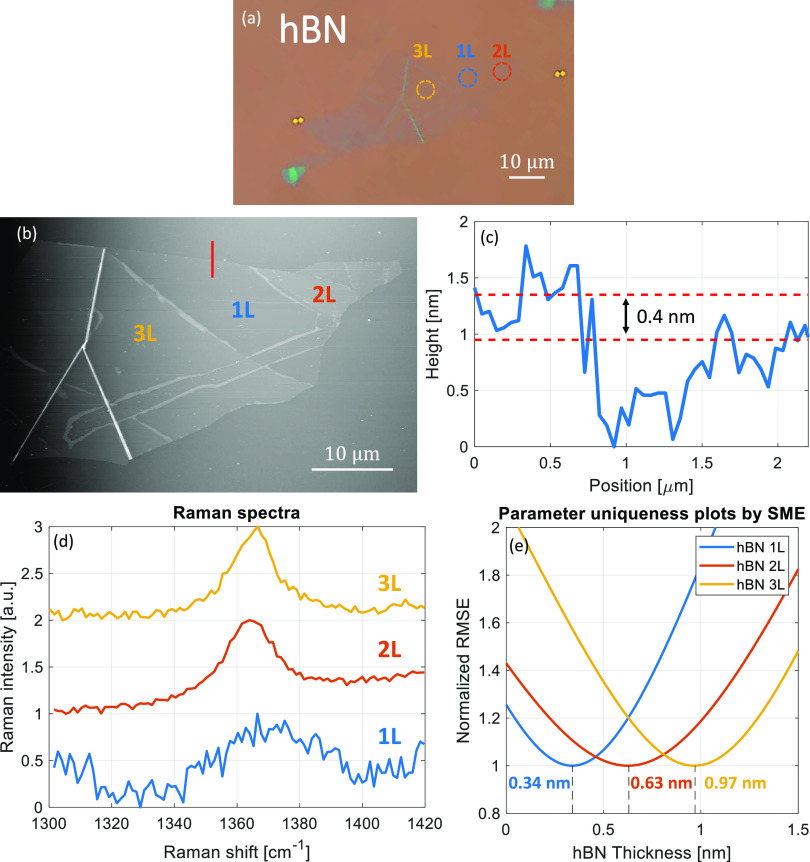
(a) Optical microscope image (contrast-enhanced for better visibility) of monolayer, bilayer and trilayer hBN with illustrated 5 μm diameter SME measurement spots in blue, orange and yellow, respectively. (b) AFM image of the hBN flake with the red line marking the transition from the monolayer hBN to the substrate and (c) the height profile of the red line showing a thickness of ∼0.4 nm, confirming the monolayer nature of the flake region. (d) The measured Raman spectra of the mono-, bi- and trilayer hBN. (e) The parameter uniqueness plots by the SME pointing to thickness results of 0.34 nm, 0.63 nm and 0.97 nm for monolayer, bilayer and trilayer hBN, respectively.

Among the flakes investigated, hBN holds special importance due to its high transparency (especially its monolayer) under optical microscope. An advantage of the SME over currently used methods is demonstrated by performing thickness measurements of an exfoliated hBN flake residing on a silicon substrate with 285 nm SiO_2_, as shown in Figure 4. Incidentally, the mono-, bi- and trilayers were found on a single flake at different locations as marked in the optical microscope image in Figure 4a. The optical contrast of the image has been amplified considerably using image processing tools to make the monolayer a bit more visible. However, it is to be noted that such tools are normally unavailable with a stand-alone optical microscope generally used for locating flakes, making the task rigorous. Even with such amplifications, the monolayer boundary is hardly discernible and only apparent in the AFM image in Figure 4b. The normalized and vertically displaced Raman spectra of the hBN flakes are plotted in Figure 4d. The relatively low-intensity and noisy peak centered at ∼1369 cm^–1^ is the Raman signature for monolayer hBN.^32^ Similar peak positions of bilayer and trilayer hBN between 1365 and 1366 cm^–1^ were demonstrated in the literature.^32^ These weak signals and tiny spectral shifts compared to their spectral widths inhibit Raman spectroscopy from confidently distinguishing between bi- and trilayers of hBN. In comparison, the AFM analysis performed on the monolayer hBN shows a thickness of ∼0.4 nm which is close to the reported values, as seen in Figure 4c. However, evidently, the AFM height profile for the monolayer is also noisy and thus less reliable. Comparatively, the SME provides thickness results with much better confidence, as inferred from the parameter uniqueness plots shown in Figure 4e. The SME clearly distinguishes between mono-, bi- and trilayers of hBN with thickness results in agreement with integer multiples of the monolayer thickness of 0.32 nm.^62^ These results clearly demonstrate the high sensitivity and superiority of the thickness measurements by the SME.

**Figure 5:**
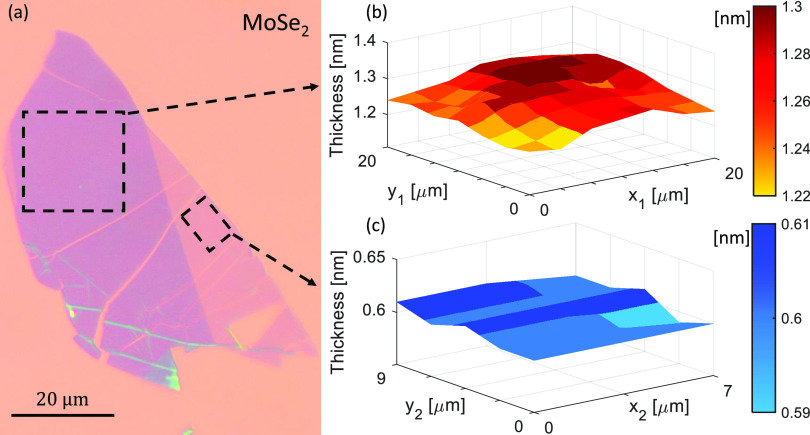
(a) Microscope image of an exfoliated MoSe_2_ flake with marked areas on bilayer (square) and monolayer (rectangle) regions. The thickness mapping results by the SME of the (b) bilayer and (c) monolayer areas with respective (*x*_1_, *y*_1_) and (*x*_2_, *y*_2_) coordinates.

To showcase the reliability and sensitivity of the SME for mapping thickness variations of flakes, thickness mapping scans on monolayer and bilayer of MoSe_2_ are performed. Figure 5a shows the optical microscope image of the exfoliated MoSe_2_ flake on 285 nm SiO_2_/Si substrate with monolayer and bilayer regions. The marked bilayer area of 20 × 20 μm^2^ is mapped with a spot size of 5 μm and a step size of 2.5 μm (49 points), and the monolayer area of 7 × 9 μm^2^ is mapped with a step size of 1 μm (15 points). The local thickness variations in the bilayer and the monolayer mapping measurements are plotted in Figure 5b,c. The mean values of 1.266 and 0.603 nm with deviations of ±0.04 nm and ±0.01 nm are obtained in the mapping measurements of bilayer and monolayer areas, respectively. In order to understand the nature of these thickness variations, repeatability measurements are performed 10 times on the same points in bilayer and monolayer areas to obtain the instrumental thickness accuracy, resulting in a deviation of ±0.005 nm for both layers. In addition, thickness variations that might be caused by the substrate oxide thickness fluctuations are found to be ±0.005 nm for both mono- and bilayer MoSe_2_ (see SI section S3). Therefore, for both mono- and bilayer mappings, the thickness variations may originate from either the flake’s landscape or from the variation of the local optical properties as a result of localized strain^68^ (reflected in the thickness results). A compressive strain can be caused by the PDMS assisted dry transfer of the flakes onto the silicon wafer and is normally estimated through shifts of the *E*_2*g*_^1^ mode in the flake’s Raman spectra.^69,70^ It is important to note that the position-dependent thickness variations caused by the lateral inhomogeneity of the flake do not influence the quantization of layer numbers. This mapping of the inhomogeneity of an exfoliated vdW flake is exclusive for spectroscopic ellipsometry, which is successfully performed on the micron-scale flake by the SME with high lateral resolution and high accuracy (via the high data acquisition rate).

Finally, the substrate-independent performance of our method is proven with a number of measurements performed on exfoliated graphene, WS_2_ and hBN flakes residing on silicon wafers with a different SiO_2_ thickness of 90 nm, showing results that are consistent and as accurate to those discussed above (see section S2 of the SI).

## Conclusions

In this work, a fast and highly sensitive Fourier imaging spectroscopic micro-ellipsometry method with high lateral resolution and data acquisition rate is used for accurate thickness mapping and thus layer number identification of various exfoliated vdW materials. The described apparatus can be seamlessly integrated as an add-on unit with any experiment involving high lateral resolution spectroscopic imaging setup or optical microscope, allowing for accurate *in situ* spectroscopic ellipsometry measurements.

Six different types of vdW materials are measured, and two different substrates are used to prove the sample- and substrate-independent performance of the proposed method. The spectroscopic micro-ellipsometer (SME) could consistently identify among mono-, bi- and trilayers of the investigated materials with sub-angstrom precision. Especially, the SME could discretely identify monolayer hBN on 285 nm SiO_2_/Si substrate, which is a challenging proposition for other characterization techniques. Repeatability measurements performed on various flakes exhibited minimal uncertainty in layer thicknesses, quantizing the layer number reliably at every iteration of measurement. Additionally, the lateral inhomogeneity of a flake comprising of mono- and bilayer areas was mapped to assess sub-angstrom thickness variations. Mapping such minute variations might provide opportunities to correlate thickness variations with local strain and with local measurements such as optical spectra and electrical transport. These results are a significant step toward an automated system capable of mapping the thickness homogeneity and allocating the exact layer number to residing flakes within a wafer area. Especially, the method can be used for locating monolayer hBN by automated scanning around other thin hBN flakes, as single-layer hBN tends to be found in their close proximity. A sensitive mapping capability of optical homogeneity with high lateral resolution should be useful for broad applications in nanotechnology and nanoscience, such as characterizing vdW devices and nanoscale metamaterials, investigating crystal structure of nanoparticles and even for probing of biological samples for variation of optical properties.

## Materials and Methods

### Sample Preparation

Various vdW materials were tape exfoliated and transferred by polydimethylsiloxane (PDMS) assisted dry transfer method onto silicon chips with a 285 nm SiO_2_ (P-type ⟨100⟩ prime grade silicon wafers from NOVA Wafers with a nominal thermal oxide thickness of 2850 Å).^63^ Based on the contrast under an optical microscope, possible candidates for mono, bi- and trilayers were identified for graphene, hBN and TMDs (MoS_2_, WS_2_, MoSe_2_ and WSe_2_), to be eventually measured with the SME for their thicknesses. Finding candidates for monolayer hBN in optical microscope was extremely challenging and required multiple iterations. The investigated flakes were pre-annealed in forming gas before micro-ellipsometry measurements to remove surface adsorbents as well as the entrapped water molecules between the flake and the substrate (150 °C for the TMDs, 300 °C for graphene and hBN). Organic adsorbates are normally trapped during PDMS assisted transfer of flakes onto silicon substrate and are squeezed into small pockets through a self-cleaning effect.^69^ These are normally reflected as bright spots or wrinkles in the AFM images. However, as can be seen from Figure 4b, the hBN flake is devoid of such features and appear to be clean, implying the annealing process has removed the organic adsorbates.

### Spectroscopic Micro-Ellipsometry Measurements

At each lateral measurement point, the SME takes four consecutive first-order images of the objective lens Fourier (back focal) plane at different polarization settings, providing spectrally and angularly resolved reflection intensity information, which is then processed to calculate the ellipsometric data of the area. In the current configuration of our micro-ellipsometer, a good signal-to-noise measurement on a 2D material flake is achieved at around 45 s. This measurement time can easily be decreased to get closer to 10 s by using a stronger light source and/or a more sensitive detector array. Our work on development of the SME^51^ gives a detailed discussion on its operation principle, data acquisition method, and instrumental performance. The ellipsometric data obtained from the substrate in the vicinity of the flake is modeled as Air/SiO_2_/Si layered structure and fitted for the oxide thickness to obtain its exact value. Then the flake data is modeled as Air/Flake/SiO_2_/Si layered structure, and the previously measured SiO_2_ thickness value is used in the model. The thin-film thickness measurement accuracy of the SME was reported to be in excellent agreement with a commercial ellipsometer in our previous work.^51^

For modeling and fitting, WVASE and CompleteEASE ellipsometry data analysis software (J.A. Woollam Co., Inc.) are used. Since just the thickness of the flake is fitted for in a simple layered structure, following building of the relevant model for the sample, the thickness fitting is nearly instantaneous, namely, negligible compared to the measurement time per position.

### Characterization with Raman Spectroscopy and Atomic Force Microscopy

The Raman measurements are performed by Renishaw InVia Confocal Raman Microscope instrument in a backscattering geometry. The excitation laser has a wavelength of 514.5 nm and the laser spot size is around 1 μm when using a 50× objective lens.

The AFM measurement is performed by the scanning probe microscope NTEGRA from NT-MDT company in tapping mode, and the results are analyzed by Nova PX software (NT-MDT).

## Supporting Information Available

Experimental data of transition metal dichalcogenide (TMD) flakes on 285 nm SiO_2_/Si substrate and of various flakes on 90 nm SiO_2_/Si, as well as a discussion on the effect of oxide thickness fluctuations of the substrate. (PDF)
