# Supplementary material for: Thickness Mapping and Layer Number Identification of Exfoliated van der Waals Materials by Fourier Imaging Micro-Ellipsometry
Source: ACS Nano. 2023 May 8;17(10):9188–96. doi: 10.1021/acsnano.2c12773 (PMC10210540; doi:10.1021/acsnano.2c12773)
Supplement: Supplementary file 1 — nn2c12773_si_002.pdf [file nn2c12773_si_002.pdf]

# Supporting Information for: Thickness Mapping and Layer Number Identification of Exfoliated van der Waals Materials by Fourier Imaging Micro-Ellipsometry

Ralfy Kenaz,*,† Saptarshi Ghosh,† Pradheesh Ramachandran,† Kenji Watanabe,‡ Takashi Taniguchi,¶ Hadar Steinberg,† and Ronen Rapaport*,†

†*Racah Institute of Physics, The Hebrew University of Jerusalem, Jerusalem 9190401, Israel*

‡*Research Center for Functional Materials, National Institute for Materials Science, 1-1 Namiki, Tsukuba 305-0044, Japan*

¶*International Center for Materials Nanoarchitectonics, National Institute for Materials Science, 1-1 Namiki, Tsukuba 305-0044, Japan*

E-mail: ralfy.kenaz@mail.huji.ac.il; ronen.rapaport@huji.ac.il

The Supporting Information (SI) elaborates the Spectroscopic Micro-Ellipsometer (SME) thickness measurements and the Raman spectra for exfoliated transition metal dichalcogenide (TMD) flakes of $MoS_2$, $WS_2$, $MoSe_2$ and $WSe_2$ residing on 285 nm $SiO_2$/Si substrates. In addition, various SME measurements of exfoliated graphene, $WS_2$ and hBN flakes on 90 nm $SiO_2$/Si substrates are demonstrated. Finally, a section highlights the effect of substrate oxide thickness fluctuations on the ellipsometric thickness measurements of the residing flakes.

# S1. $MoS_2$, $WS_2$, $MoSe_2$ and $WSe_2$ results

Figures S1-S4(a) show the optical microscope images of mono-, bi- and trilayer flakes of $MoS_2$, $WS_2$, $MoSe_2$ and $WSe_2$, respectively. Figures S1-S4(b) are the parameter uniqueness plots by the SME from the spectroscopic micro-ellipsometry measurements performed on the illustrated 5 $\mu m$ diameter areas on mono- (blue), bi- (orange) and trilayer (yellow) regions; resulting in thickness values of the measured flakes. All thickness results are within the tolerance limit from integer multiples of the monolayer thicknesses of 0.6-0.7 nm for the TMDs,[1–5] as mentioned in the paper. Finally, Figures S1-S4(c) plot the measured Raman spectra of the flakes in normalized and vertically displaced forms for clarity, where the excitation wavelength of the laser is 514.5 nm.

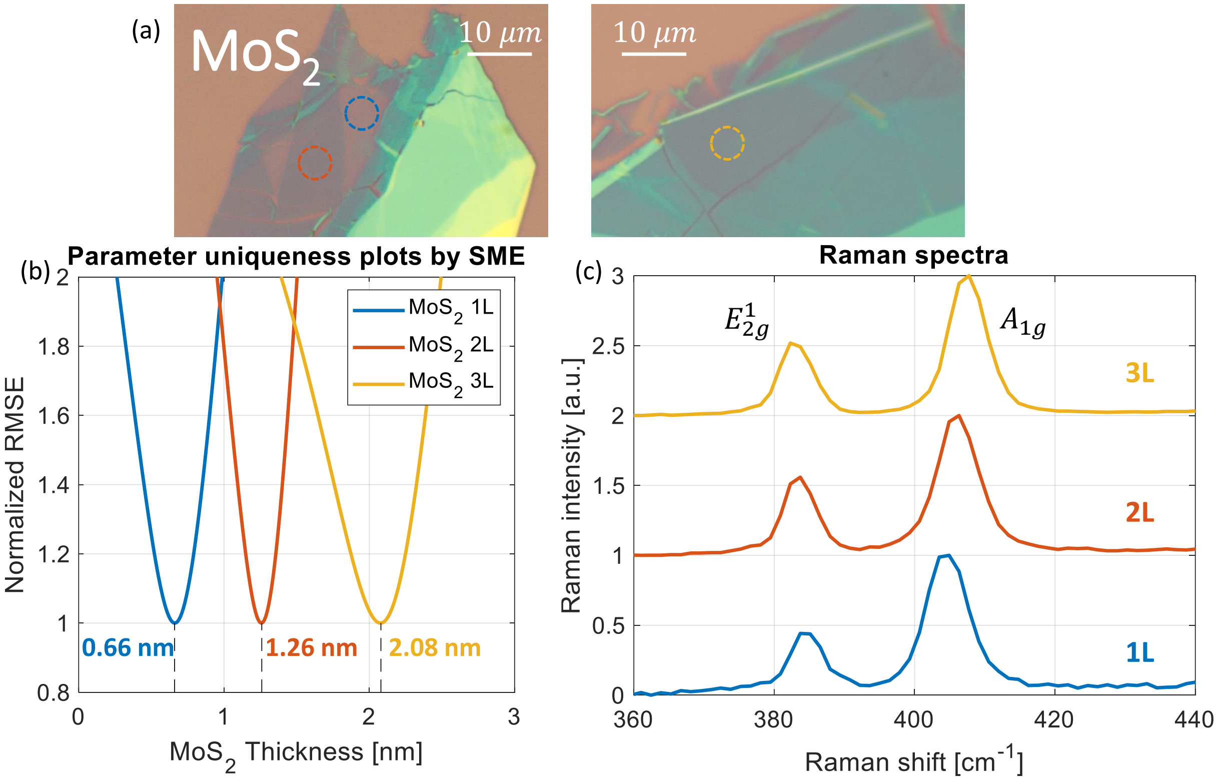


Figure S1: (a) Optical microscope images of monolayer, bilayer and trilayer $MoS_2$ with illustrated 5 $\mu m$ diameter SME measurement spots in blue, orange and yellow, respectively. (b) The parameter uniqueness plots by the SME pointing to thickness results of 0.66 nm, 1.26 nm and 2.08 nm for monolayer, bilayer and trilayer $MoS_2$, respectively. (c) The measured Raman spectra of the same flakes.

The measured Raman spectra of the $MoS_2$ flakes are plotted in Figure S1(c). The frequency difference ($\Delta\omega$) between the two Raman modes of $E^1_{2g}$ and $A_{1g}$ for $MoS_2$ gives a strong indication for the number of layers. It has been shown that the $\Delta\omega$ for $MoS_2$ monolayer is $\sim$3 $cm^{-1}$ smaller than its bilayer, and the bilayer is $\sim$1.5 $cm^{-1}$ smaller than the trilayer.[6] This difference follows a decreasing trend until 6-layers and stabilizes at bulk state.[6] In Figure S1(c), the $\Delta\omega$ between the peaks are 19.8 $cm^{-1}$, 22.7 $cm^{-1}$ and 24.3 $cm^{-1}$ for mono-, bi- and trilayer, respectively. This accounts to $\sim$3 $cm^{-1}$ and $\sim$1.5 $cm^{-1}$ difference between 1L-2L and 2L-3L respectively, confirming the mono-, bi- and trilayer natures of the $MoS_2$ flakes.

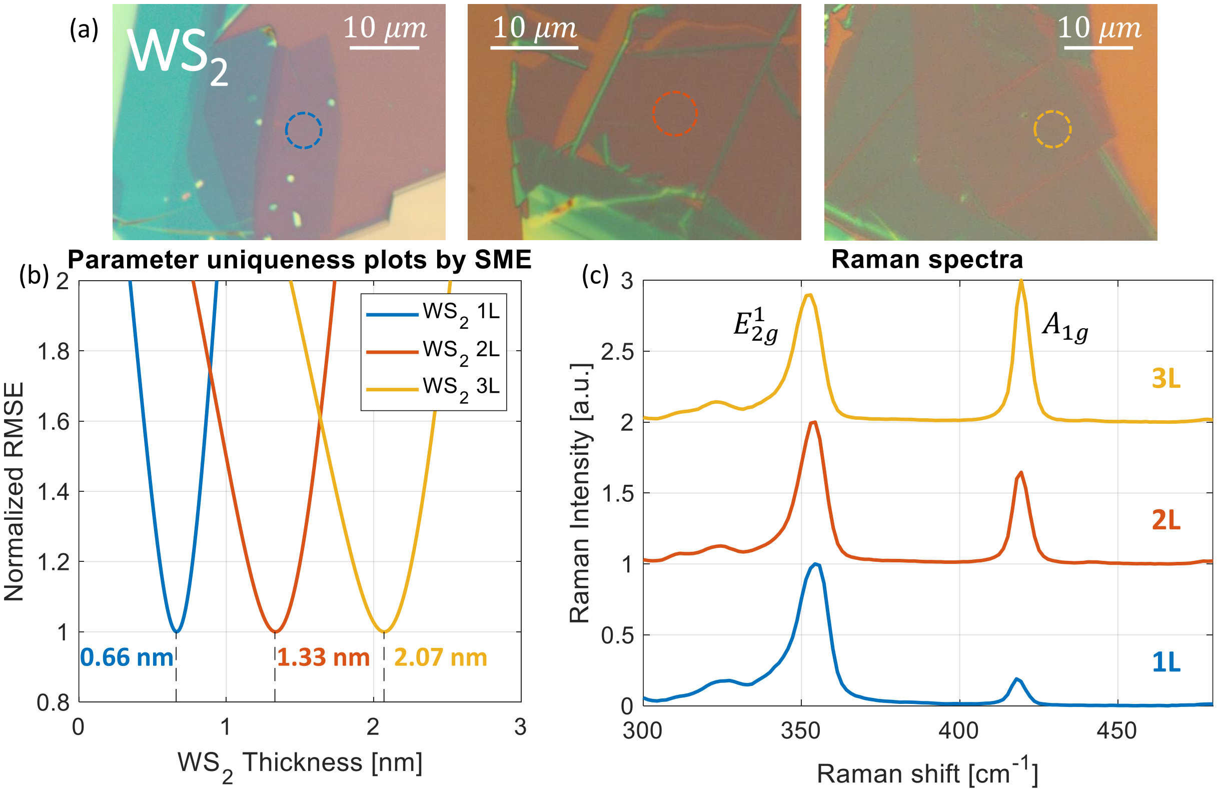


Figure S2: (a) Optical microscope images of monolayer, bilayer and trilayer $WS_2$ with illustrated 5 $\mu m$ diameter SME measurement spots in blue, orange and yellow, respectively. (b) The parameter uniqueness plots by the SME pointing to thickness results of 0.66 nm, 1.33 nm and 2.07 nm for monolayer, bilayer and trilayer $WS_2$, respectively. (c) The measured Raman spectra of the same flakes.

The measured Raman spectra of the $WS_2$ flakes are plotted in Figure S2(c). As the Raman fingerprints for layer numbers of $WS_2$ flakes, the peak intensity ratio and frequency difference of the $E^1_{2g}$ and $A_{1g}$ modes at $\sim$350 $cm^{-1}$ and $\sim$420 $cm^{-1}$, respectively, are taken

into consideration. Peak intensity ratios of 4.5, 1.5 and 0.8 are received for the mono-, bi- and trilayer $WS_2$ measured in this work, respectively. A peak intensity ratio that is larger than 2 has been reported to be an exclusive signature for monolayer $WS_2$, followed by intensity ratios of roughly 1 and 0.7 for bilayer and trilayer, respectively.[7,8] In addition, the frequency differences between the peaks show an increasing trend as $\Delta\omega = 63.7$ cm$^{-1}$, 65.8 cm$^{-1}$ and 67.2 cm$^{-1}$ from mono- to trilayer, which is in good agreement with the literature.[8]

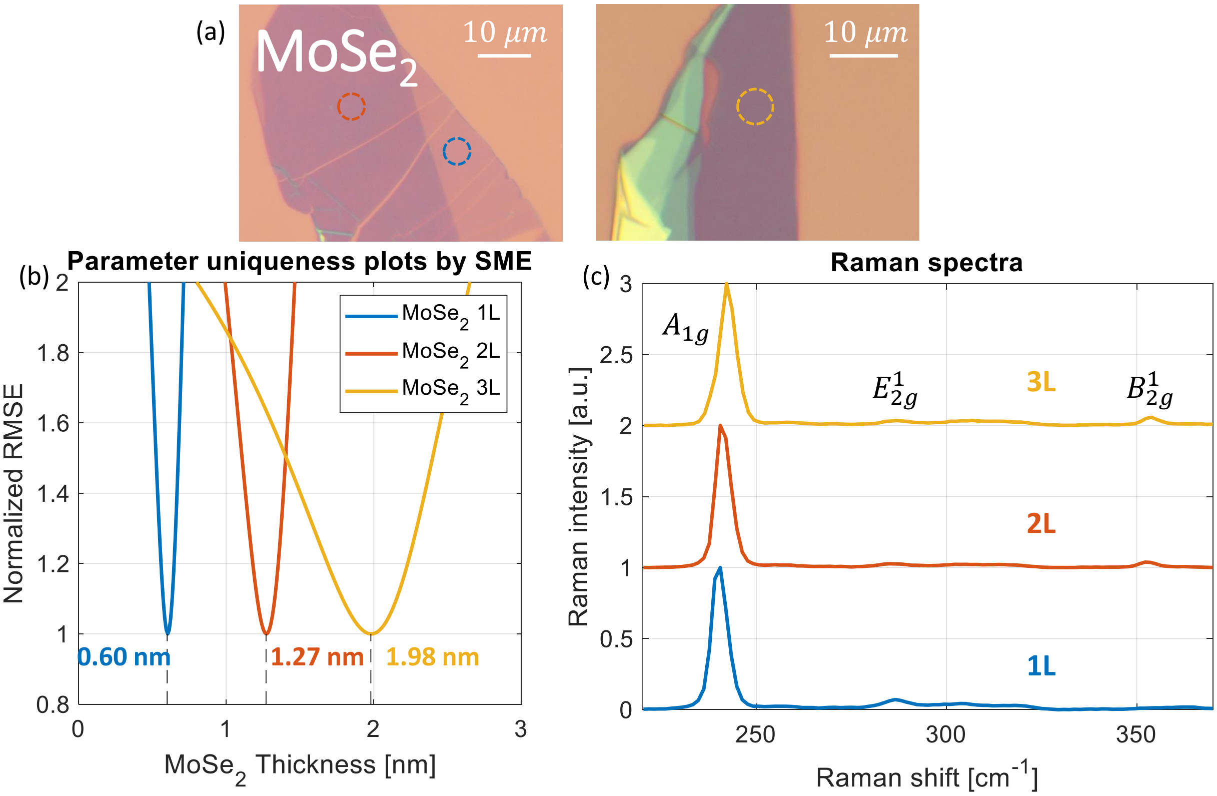


Figure S3: (a) Optical microscope images of monolayer, bilayer and trilayer $MoSe_2$ with illustrated 5 $\mu m$ diameter SME measurement spots in blue, orange and yellow, respectively. (b) The parameter uniqueness plots by the SME pointing to thickness results of 0.60 nm, 1.27 nm and 1.98 nm for monolayer, bilayer and trilayer $MoSe_2$, respectively. (c) The measured Raman spectra of the same flakes.

The measured Raman spectra of the $MoSe_2$ flakes are plotted in Figure S3(c). The out-of-plane $A_{1g}$ mode peak is found at 240.5 cm$^{-1}$ for monolayer $MoSe_2$, slightly shifting towards higher wavenumbers for bi-, and trilayers. The absolute intensity of this peak is highest for the bilayer (not shown here due to normalization). The lower wavenumber side of the $A_{1g}$

mode in the trilayer shows some broadening, probably due to existence of another vibrational component which is not resolved by the used Raman instrument. The weak in-plane $E^1_{2g}$ mode is found around 287 cm$^{-1}$ for monolayer and 286 cm$^{-1}$ for bilayer. The $B_{2g}$ mode at 353 cm$^{-1}$ does not exist for the monolayer and has the highest intensity for the bilayer. All these findings agree with the literature[9] and confirm the monolayer, bilayer and trilayer natures of the flakes.

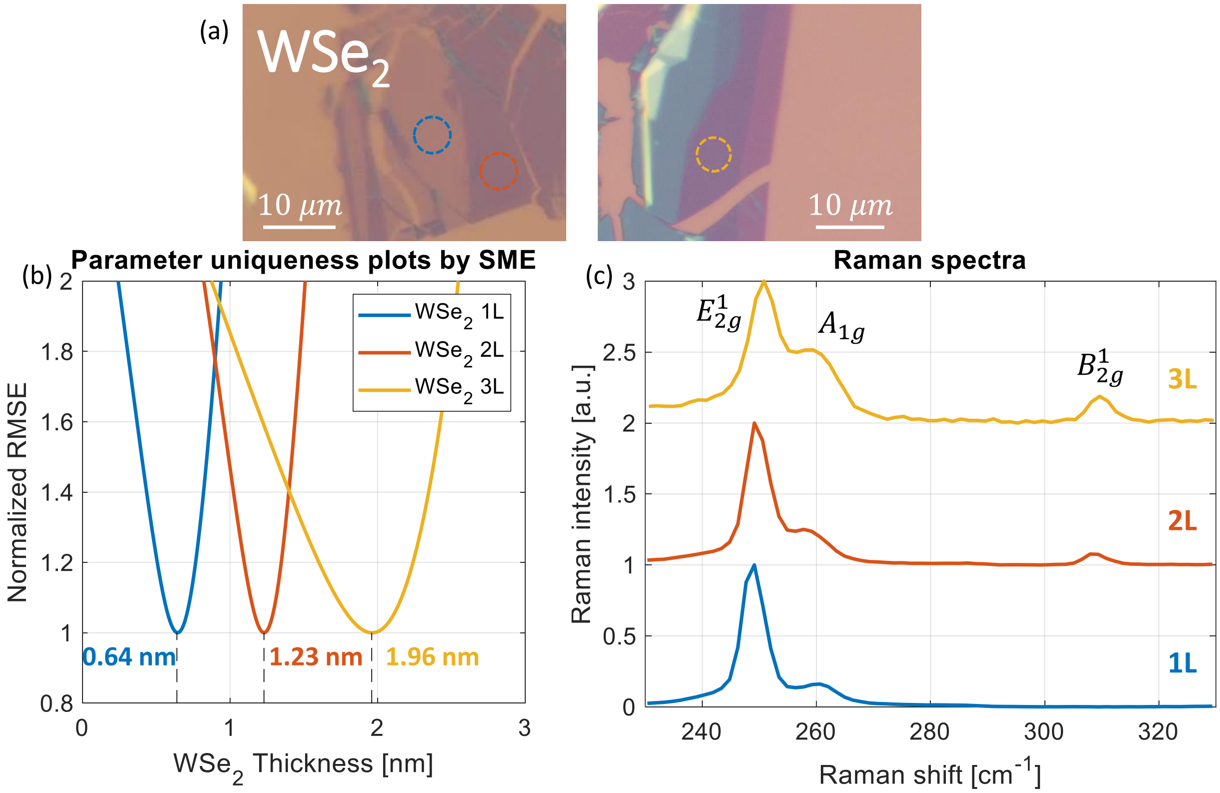


Figure S4: (a) Optical microscope images of monolayer, bilayer and trilayer $WSe_2$ with illustrated 5 $\mu m$ diameter SME measurement spots in blue, orange and yellow, respectively. (b) The parameter uniqueness plots by the SME pointing to thickness results of 0.64 nm, 1.23 nm and 1.96 nm for monolayer, bilayer and trilayer $WSe_2$, respectively. (c) The measured Raman spectra of the same flakes.

The measured Raman spectra of the $WSe_2$ flakes are plotted in Figure S4(c). The main vibrational mode $E^1_{2g}$ is located around 249 cm$^{-1}$ for the monolayer $WSe_2$, showing a slight blueshift with increasing number of layers. The intensity of this peak for the monolayer is the highest, being ∼2.5 times of the bilayer and ∼25 times of the trilayer (not shown here

due to normalization). The $B^1_{2g}$ mode around 309 $cm^{-1}$ is not existent for the monolayer and shows the highest intensity counts for the bilayer. These Raman signatures concur with the literature[8,9] and confirm that the measured flakes are indeed mono-, bi- and trilayers of $WSe_2$.

## S2. Measurements on a different substrate

A number of measurements of various flakes are performed on silicon wafer substrates with 90 nm $SiO_2$. Graphene, $WS_2$ and hBN are chosen to cover the whole range of materials

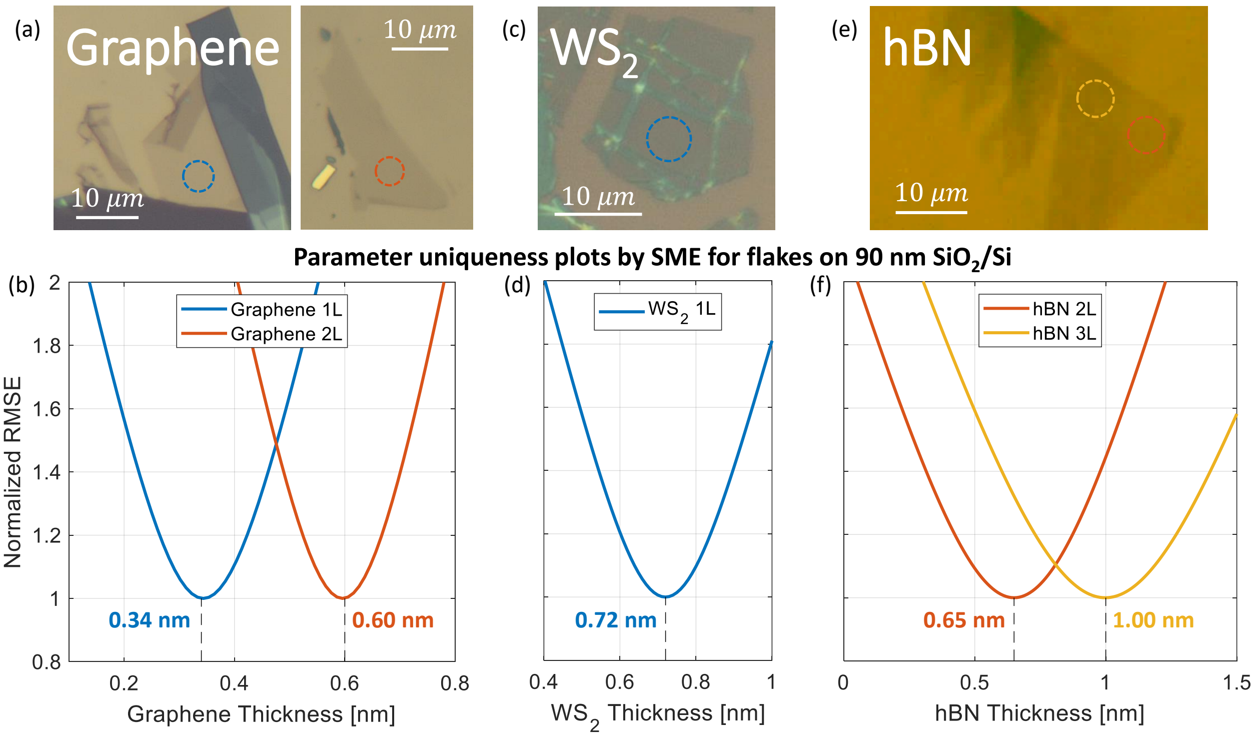


Figure S5: A number of additional flake measurements repeated on Si wafers with 90 nm of $SiO_2$ for the sake of demonstrating the substrate-independent performance of the proposed method. (a) Graphene monolayer and bilayer optical microscope images with illustrated 5 $\mu m$ diameter SME measurement spots and (b) their parameter uniqueness plots pointing to thickness results of 0.34 nm and 0.60 nm for the monolayer and bilayer, respectively. (c) $WS_2$ monolayer optical microscope image and (d) its parameter uniqueness plot pointing to thickness result of 0.72 nm. (e) hBN bilayer and trilayer optical microscope images (contrast-enhanced for better visibility) and (f) their parameter uniqueness plots pointing to thickness results of 0.65 nm and 1.00 nm for the bilayer and trilayer, respectively.

discussed in the paper. Figure S5 shows identical performance as obtained on silicon wafers with 285 nm $SiO_2$, proving the substrate-independent performance of the SME.

The Raman spectra of these flakes are also measured, resulting in very similar responses as demonstrated for the same materials on silicon wafers with 285 nm $SiO_2$.

## S3. Effect of substrate oxide thickness fluctuations

The thickness fluctuations of the substrate oxide layer was measured on a same type of silicon wafer in our recent paper.[10] While scanning an area of $35 \times 35$ $\mu m^2$ with a 5 $\mu m$ spot size and a 5 $\mu m$ step size, the fluctuation in the oxide thickness was measured to be $\pm 0.17$ nm. Repeatability measurements performed on the same spot returned a value of $\pm 0.04$ nm, thus totalling to a $\pm 0.21$ nm maximal fluctuation in the oxide thickness. In the models of all the flake measurements, this value was added to and subtracted from the corresponding oxide thicknesses below the flakes, and the flake thicknesses were fitted for. The resulting maximum flake thickness variations are plotted in Figure S6.

The error bars in Figure S6 show the largest variations in thicknesses of the hBN flakes, followed by graphene, and finally TMDs. Since hBN has the lowest ellipsometric contrast (showing high similarity in optical properties with the below oxide, and therefore is near-transparent above it), a fluctuation of $\pm 0.21$ nm in the oxide thickness translates into $\sim \pm 0.05$ nm in the residing hBN flake thickness. With graphene, this number decreases to $\pm 0.02$ nm, and for TMDs (the materials with the highest ellipsometric contrast), it is between $\pm 0.02$ nm and $\pm 0.005$ (mostly $\pm 0.01$ nm). For all cases, it is safe to say that the substrate oxide thickness fluctuations do not interfere with the mentioned method's capability to accurately distinguish between mono-, bi- and trilayers of vdW materials.

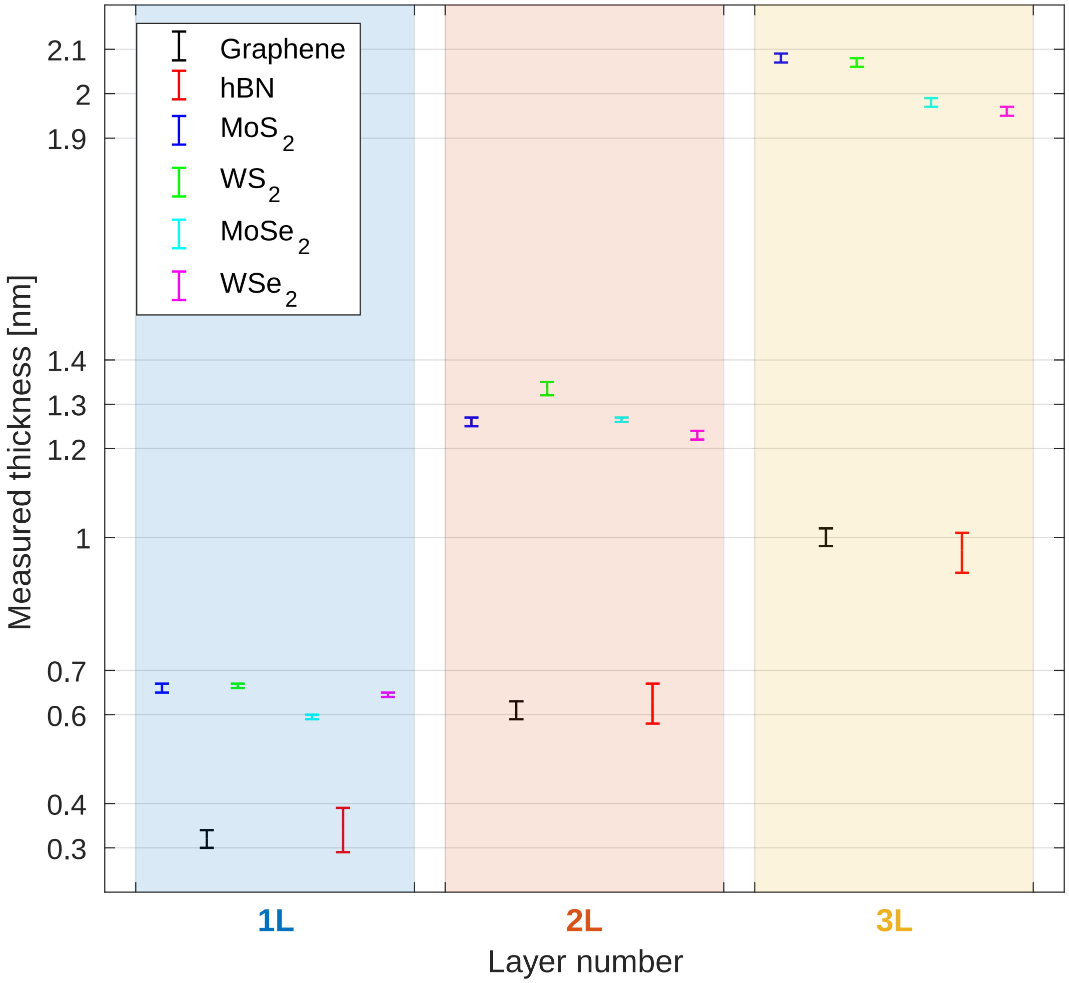


Figure S6: The maximum variations (error bars) in all flake thicknesses resulting from ±0.21 nm maximum thickness fluctuation of the oxide layer below.

# References

1. Benameur, M. M.; Radisavljevic, B.; Héron, J. S.; Sahoo, S.; Berger, H.; Kis, A. Visibility of Dichalcogenide Nanolayers. *Nanotechnology* **2011**, *22*, 125706.

2. Sahin, H.; Tongay, S.; Horzum, S.; Fan, W.; Zhou, J.; Li, J.; Wu, J.; Peeters, F. M. Anomalous Raman Spectra and Thickness-Dependent Electronic Properties of $WSe_2$. *Physical Review B - Condensed Matter and Materials Physics* **2013**, *87*, 165409.

3. Kim, H. C.; Kim, H.; Lee, J. U.; Lee, H. B.; Choi, D. H.; Lee, J. H.; Lee, W. H.; Jhang, S. H.; Park, B. H.; Cheong, H.; Lee, S. W.; Chung, H. J. Engineering Optical

and Electronic Properties of $WS_2$ by Varying the Number of Layers. *ACS Nano* **2015**, *9*, 6854–6860.

4. He, Y.; Sobhani, A.; Lei, S.; Zhang, Z.; Gong, Y.; Jin, Z.; Zhou, W.; Yang, Y.; Zhang, Y.; Wang, X.; Yakobson, B.; Vajtai, R.; Halas, N. J.; Li, B.; Xie, E.; Ajayan, P. Layer Engineering of 2D Semiconductor Junctions. *Advanced Materials* **2016**, *28*, 5126–5132.

5. Qin, F.; Liu, B.; Zhu, L.; Lei, J.; Fang, W.; Hu, D.; Zhu, Y.; Ma, W.; Wang, B.; Shi, T.; Cao, Y.; Guan, B. o.; Qiu, C. w.; Lu, Y.; Li, X. $\pi$-Phase Modulated Monolayer Supercritical Lens. *Nature Communications* **2021**, *12*, 1–9.

6. Lee, C.; Yan, H.; Brus, L. E.; Heinz, T. F.; Hone, J.; Ryu, S. Anomalous Lattice Vibrations of Single- and Few-Layer $MoS_2$. *ACS Nano* **2010**, *4*, 2695–2700.

7. Berkdemir, A.; Gutiérrez, H. R.; Botello-Méndez, A. R.; Perea-López, N.; Elías, A. L.; Chia, C. I.; Wang, B.; Crespi, V. H.; López-Urías, F.; Charlier, J. C.; Terrones, H.; Terrones, M. Identification of Individual and Few Layers of $WS_2$ Using Raman Spectroscopy. *Scientific Reports* **2013**, *3*, 1–8.

8. Zeng, H.; Liu, G. B.; Dai, J.; Yan, Y.; Zhu, B.; He, R.; Xie, L.; Xu, S.; Chen, X.; Yao, W.; Cui, X. Optical Signature of Symmetry Variations and Spin-Valley Coupling in Atomically Thin Tungsten Dichalcogenides. *Scientific Reports* **2013**, *3*, 1–5.

9. Tonndorf, P.; Schmidt, R.; Böttger, P.; Zhang, X.; Börner, J.; Liebig, A.; Albrecht, M.; Kloc, C.; Gordan, O.; Zahn, D. R. T.; Michaelis de Vasconcellos, S.; Bratschitsch, R. Photoluminescence Emission and Raman Response of Monolayer $MoS_2$, $MoSe_2$, and $WSe_2$. *Optics Express* **2013**, *21*, 4908.

10. Kenaz, R.; Rapaport, R. Mapping Spectroscopic Micro-Ellipsometry with Sub-5 Microns Lateral Resolution and Simultaneous Broadband Acquisition at Multiple Angles. *Review of Scientific Instruments* **2023**, *94*, 023908.
